# ‘Advocacy groups are the connectors’: Experiences and contributions of rare disease patient organization leaders in advanced neurotherapeutics

**DOI:** 10.1111/hex.13625

**Published:** 2022-10-28

**Authors:** Christina Q. Nguyen, Didu Kariyawasam, Kristine Alba‐Concepcion, Sarah Grattan, Kate Hetherington, Claire E. Wakefield, Susan Woolfenden, Russell C. Dale, Elizabeth E. Palmer, Michelle A. Farrar

**Affiliations:** ^1^ Discipline of Paediatrics and Child Health, School of Clinical Medicine, Faculty of Medicine and Health UNSW Sydney Sydney New South Wales Australia; ^2^ Department of Neurology Sydney Children's Hospital Network Randwick New South Wales Australia; ^3^ Behavioural Sciences Unit, Kids Cancer Centre Sydney Children's Hospital Randwick New South Wales Australia; ^4^ Sydney Institute Women Children and their Families Sydney New South Wales Australia; ^5^ Children's Hospital at Westmead Clinical School University of Sydney Westmead New South Wales Australia; ^6^ Centre for Clinical Genetics Sydney Children's Hospital Network Randwick New South Wales Australia

**Keywords:** advanced therapeutics, paediatric neurology, patient advocacy, precision medicine, rare diseases

## Abstract

**Introduction:**

Biomedical progress has facilitated breakthrough advanced neurotherapeutic interventions, whose potential to improve outcomes in rare neurological diseases has increased hope among people with lived experiences and their carers. Nevertheless, gene, somatic cell and other advanced neurotherapeutic interventions carry significant risks. Rare disease patient organizations (RDPOs) may enhance patient experiences, inform expectations and promote health literacy. However, their perspectives are understudied in paediatric neurology. If advanced neurotherapeutics is to optimize RDPO contributions, it demands further insights into their roles, interactions and support needs.

**Methods:**

We used a mixed‐methodology approach, interviewing 20 RDPO leaders representing paediatric rare neurological diseases and following them up with two online surveys featuring closed and open‐ended questions on advanced neurotherapeutics (19/20) and negative mood states (17/20). Qualitative and quantitative data were analysed using thematic discourse analysis and basic descriptive statistics, respectively.

**Results:**

Leaders perceived their roles to be targeted at educational provision (20/20), community preparation for advanced neurotherapeutic clinical trials (19/20), information simplification (19/20) and focused research pursuits (20/20). Although most leaders perceived the benefits of collaboration between stakeholders, some cited challenges around collaborative engagement under the following subthemes: conflicts of interest, competition and logistical difficulties. Regarding neurotherapeutics, RDPO leaders identified support needs centred on information provision, valuing access to clinician experts and highlighting a demand for co‐developed, centralized, high‐level and understandable, resources that may improve information exchange. Leaders perceived a need for psychosocial support within themselves and their communities, proposing that this would facilitate informed decision‐making, reduce associated psychological vulnerabilities and maintain hope throughout neurotherapeutic development.

**Conclusion:**

This study provides insights into RDPO research activities, interactions and resource needs. It reveals a demand for collaboration guidelines, central information resources and psychosocial supports that may address unmet needs and assist RDPOs in their advocacy.

**Patient or Public Contribution:**

In this study, RDPO leaders were interviewed and surveyed to examine their perspectives and roles in advanced neurotherapeutic development. Some participants sent researchers postinterview clarification emails regarding their responses to questions.

## 1 INTRODUCTION

Worldwide, the average defining prevalence threshold for a rare disease is between 40 and 50 cases per 100,000 people.^1^ Of all rare diseases, 72%–80% have genetic aetiologies, 75% exhibit neurological signs and 70% demonstrate exclusively paediatric onset.^2^, ^3^ Although paediatric rare neurological diseases (RNDs) cite low prevalence individually, they collectively constitute a large population, whose health needs are frequently complicated, severe and unmet.^4^, ^5^, ^6^, ^7^

Rare disease patient organizations (RDPOs) serve multidimensional roles, responding to patient and family needs and maintaining an active presence in biomedical discourse.^5^, ^8^, ^9^, ^10^ Through educational tool development, service coordination, community building and research funding, they can expedite patient therapeutic access and, in tandem with treatment innovations, optimize outcomes.^10^, ^11^, ^12^ As mediators, they may facilitate interactions between patients, families, researchers and industry to improve information exchange and promote health and research literacy.^13^, ^14^, ^15^, ^16^ In doing so, they may advise therapeutic endpoints based on community priorities.^13^, ^14^, ^15^, ^16^ Given their access to widely dispersed patients, RDPOs are uniquely positioned to accumulate genotypic and phenotypic data.^17^ Thus, they frequently offer support in project promotion, study recruitment, biobank construction and patient registry formation.^17^, ^18^, ^19^

Echoing the momentum of biomedical advancements, RDPO roles continue to expand.^20^, ^21^ As rare disease research benefits from global development, regulatory approval and governmental subsidisation of innovative breakthrough therapies, RDPOs have become assertive research collaborators.^22^, ^23^, ^24^, ^25^, ^26^, ^27^, ^28^, ^29^, ^30^ In fact, studies have embraced the person‐centred research model, calling for active patient engagement throughout rare disease academia.^31^, ^32^ The roles RDPOs play in rare disease information resource development have appeared frequently throughout literature; however, a question remains as to how RDPOs, themselves, access reliable information, in a sphere where publicly accessible, expert information is scarce.^33^ What is more, qualitative and mixed methodology studies have reported heterogeneity across RDPO experiences, citing psychosocial themes of frustration and disillusionment alongside hope and empowerment.^8^, ^18^ While critical to promoting patient care in this dynamically evolving arena, RDPO research contributions, experiences and perspectives remain underexplored, with most studies analysing them generally rather than in medically specialized fields.^34^

Furthermore, advanced neurotherapeutics—namely, gene, somatic cell, tissue‐engineered and combined products—for paediatric RNDs encounter challenges distinct from other rare diseases.^35^, ^36^ Often experimental, they confront unique ethical dilemmas surrounding consent, autonomy, parental capacity and quality‐versus‐prolongation‐of‐life.^37^ Moreover, advanced neurotherapeutic clinical trials encounter operational complexities of transparency, equity, limited resources and sample size restrictions.^35^ In this novel therapeutic domain, there is an urgency to understand RDPO's scope of practice, interstakeholder partnerships, information requirements and psychosocial needs. Greater insights into these fields are imperative if clinical development is to maximally benefit from RDPO expertise.^38^

Due to the relative paucity of original research, a mixed methodology approach was selected to analyse the complex perspectives of RDPO leaders operating within advanced neurotherapeutics. This study's research aims were to evaluate:

1. The perspectives of RDPO leaders regarding their roles and interactions in advanced neurotherapeutic development.
2. The perspectives of RDPO leaders regarding their own information and psychosocial support needs, and those of their communities, in this field.

We hypothesized that RDPO leaders would outline wide organizational responsibilities and interactions—especially with industry, researchers and patient families—and perceive current information and psychosocial supports as insufficient or poorly accessible. We envisaged that our findings would inform future educational resources, whose distribution among RDPOs, parents and clinicians could assist decision‐making around advanced neurotherapeutics.

## 2 METHODS

### 2.1 Participants and recruitment strategy

RDPO leaders were eligible if they represented patient families affected by paediatric RNDs and had sufficient English language skills to partake in discussion without an interpreter. Purposive sampling and snowballing via inter‐RDPO introductions were adopted throughout recruitment. To maximize heterogeneity and characterize the spectrum of perspectives, all partner organizations listed on the Rare Voices Australia (RVA) website; large RDPO alliances throughout Europe and North America; and other RDPOs known to researchers through established health partnerships were invited to participate.^39^ As Australia's national peak body on rare diseases, RVA offered a catalogue of partner RDPOs for sampling.

An invitation letter, consent form and information sheet were emailed to the chief executive officers (CEOs) of all eligible RDPOs. Invitees were contacted a maximum of two times to confirm email receipt, gauge interest in participation and address study queries. Interviews were conducted with either invitees themselves or with their nominated organizational representatives. Participants were informed of the study aims and protocols around confidential reporting.

### 2.2 Data collection

Written informed consent was obtained from all participants before interviews, which were conducted via Zoom Meetings (Zoom Video Communications Inc.). All interviews were coordinated, video‐recorded and transcribed verbatim by the first author. To clarify meaning, interpretations of responses were relayed to participants throughout interviews and postinterview clarification emails were sent. A review of the literature and specialist consultation (psychology, paediatric neurology) informed interview guide development. This guide was used to elicit participant perspectives on RDPO roles. It featured open‐ended questions on participants' understandings and expectations of advanced neurotherapeutics—namely, benefits, risks, alternatives, information access and collaboration. Question phraseology and sequencing remained flexible to promote open discussion (Supporting Information: Appendices A and B). Each interview was conducted once, utilizing the same interview guide, and involved one participant, interviewer and facilitator. Throughout each interview, the facilitator served as an objective observer and information source around this study project, addressing participants' queries and ensuring that the interviewer did not introduce loaded questions and wording bias.

### 2.3 Postinterview study measures

Within 24 h postinterview, two surveys were distributed to participants via emailed hyperlinks and stored in Qualtrics XM. Due to their notably distinct subject matters, these surveys were circulated as separate hyperlinks and stored independently for convenient data management. A custom‐designed survey (Questionnaire 1) collected participant demographic details and opinions regarding advanced neurotherapeutics. It incorporated ranking scales, open‐ended questions and 5‐point Likert scales (1 ‘strongly disagree’ to 5 ‘strongly agree’, 1 ‘disagree’ to 5 ‘agree’, 1 ‘not at all’ to 5 ‘very’) (Supporting Information: Appendix C). A second survey used Depression, Anxiety, Stress Scale‐21 Items (DASS‐21) to assess participants' depression, anxiety and stress symptoms over the past week.^40^ Each survey question analysed one of three negative mood states and integrated four‐point frequency scales (0 ‘did not apply to me at all’ to 3 ‘applied to me very much or most of the time’) (Supporting Information: Appendix D). As per Lovibond and Lovibond,^40^ responses were allocated numerical values, arithmetically summed and compared with recommended cut‐off ranges for normal, mild, moderate, severe and extremely severe syndromes (Supporting Information: Appendix D). Surveys were later matched with their corresponding participant interviews.

### 2.4 Data analysis

This study utilized a triangulation design, through which qualitative and quantitative data were synthesized. Specifically, convergent triangulation was implemented, with qualitative and quantitative data collected and analysed separately but concurrently.^41^ Qualitative and quantitative findings were merged on a construct‐by‐construct basis, such that quantitative scales were linked with qualitative themes.^41^ In this way, data merging was ‘qualitatively driven’ or guided by qualitative results.^42^

Using the conceptual framework by Miles et al.,^43^ interview transcripts were analysed in four phases. First, transcripts were reviewed and annotated for salient material by six individual researchers. Next, transcripts underwent group discussion, during which narrative summaries and major themes were developed for each interview. Last, all salient results were synthesized into a thematic coding tree. Using NVivo 12 Pro, the first two transcripts were coded by the first author, whose results were confirmed by a secondary coder. Once interrater reliability utilizing percentage agreement methodology revealed a high degree of concordance (97%), the first author independently coded the remaining transcripts line‐by‐line. All researchers reviewed coding and convened throughout data collection and analysis via weekly meetings. The quotient of leaders exploring each qualitative theme was calculated before data merging.

Quantitative and demographic data were analysed using IBM SPSS version 27 and represented as frequencies and descriptive statistics. There were three types of 5‐point Likert scales. For frequencies, the first was regrouped: ‘strongly disagree and disagree’, ‘neither agree nor disagree’ and ‘agree and strongly agree’. The second was regrouped: ‘disagree and somewhat disagree’, ‘neither agree nor disagree’ and ‘somewhat agree and agree’. The third was regrouped: ‘not at all and not very’, ‘neither’ and ‘somewhat and very’.

## 3 RESULTS

### 3.1 Demographics

Of 41 eligible RDPO leaders, 20 consented into the study, yielding an overall response rate of 49% (Supporting Information: Appendix E). Only one representative from each RDPO participated in an interview. Each of the 20 interviews lasted between 35 and 73 min, with a mean time of 50 min. Postinterview, 19/20 completed Questionnaire 1 and 17/20 completed the DASS‐21. Participants came from diverse sociocultural backgrounds (Table 1). All had received tertiary education and at least 13/20 were either working full‐time or part‐time for their RDPOs. Participating RDPOs were representatives from a range of paediatric RNDs, including 10/20 neurodevelopmental, 4/20 neuromuscular, 4/20 neurodegenerative and 2/20 undiagnosed or nonspecifically rare conditions. Of the cohort, 17/20 were based in Australia, 1/20 in the United States, 1/20 in the United Kingdom and 1/20 in Egypt. Notably, 12/20 of the RDPO leaders had at least one child affected by paediatric RND. Of these participants, two thirds (8/20) served as CEOs or board members within their RDPOs. Likewise, all RDPO leaders who founded their RDPOs (6/20) had at least one affected child.

**Table 1:** Participant characteristics

Characteristic	*n* = 20
Age range (years)	
21–30	1
31–40	3
41–50	12
51–60	2
61–70	1
71–80	1
Gender	
Female	14
Male	6
Religion	
Christianity	7
Judaism	1
No religion	11
Undisclosed	1
Relationship status	
Currently married or in de facto relationship	16
Separated or divorced	2
Widowed	1
Other	1
First language	
English	18
Other	2
Highest attained level of education	
Certificate/diploma	3
University degree	6
Postgraduate degree	11
Number of children with rare neurological disease	
0	8
1	10
2	2
Current role within organization	
Board member	2
Chief executive officer	10
Founder (another role may be held concurrently)	6
President	1
Head of research	3
Support coordinator	1
General member	1
Employment status within organization	
Full‐time	8
Part‐time	5
Undisclosed	7
Organization's focus disease or group of diseases	
Neuromuscular	4
Duchenne muscular dystrophy	
Hereditary spastic paraplegia	
Muscular dystrophy	
Spinal muscular atrophy	
Neurodevelopmental	10
Angelman syndrome	
Cerebral palsy	
*CLCN4*‐related condition	
*FOXG1*‐related condition	
*IQSEC2*‐related condition	
Kleefstra syndrome	
Neurofibromatosis type 1	
Tuberous sclerosis	
*SCN2A*‐related condition	
*SYNGAP1*‐related condition	
Neurodegenerative	4
Childhood dementia	
Leukodystrophy	
Neuronal ceroid lipofuscinoses	
Mitochondrial disease	
Undiagnosed or nonspecifically rare	2
Rare diseases	
Undiagnosed disorders	

**Figure 1:**
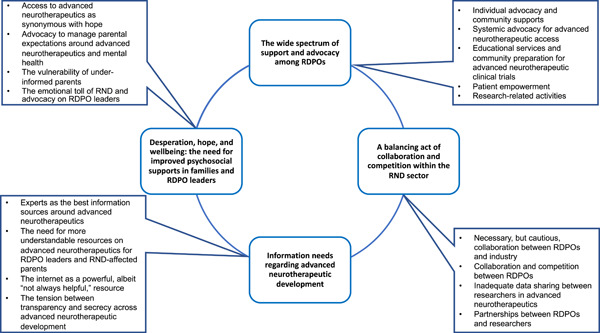
A graphical summary of themes around the perspectives, relationships and experiences of RDPO leaders in advanced neurotherapeutic development. Themes are bolded and centrally located. Subthemes are listed as bullet points in outside boxes. RDPO, rare disease patient organization; RND, rare neurological disease.

Qualitative and quantitative findings revealed several salient themes (Figure 1).

### 3.2 Perceived roles of RDPOs in advanced neurotherapeutics

All leaders framed their advocacy around notions of altruism and described ventures within specific advanced neurotherapeutic areas. They reported a plethora of perceived roles, from facilitating patient family education and community networking to research.

### 3.3 The wide spectrum of support and advocacy among RDPOs

Notably, all leaders reported offering educational services and resources to patients, clinicians, governments and other stakeholders. Almost all (19/20) RDPO leaders reported preparing their communities for advanced neurotherapeutic clinical trials. Specifically, they expressed a responsibility to educate families on therapeutic efficacy, benefit‐risk profile, administration method and mechanism of action, as well as trial eligibility, accessibility and structure.

From the cohort, 19/20 emphasized the role of RDPOs in filtering and simplifying complex information for families. Indeed, 11/20 spontaneously declared the significance of their organization's educative responsibilities in patient empowerment. One leader stated:

It might be just making sure that we can communicate all the stuff we've been talking about well to people, so that they feel like they've got the information to make the right choices, the right decisions … so they say, ‘we can participate in a trial with confidence, or a drug that's been approved, because we understand it’. (A1)

All leaders described engagement in research‐related activities, such as grant funding, agenda‐setting and data collection. Within this framework, interstakeholder connection (19/20) emerged consistently as a strong subtheme in the perceived role of RDPOs. As one leader declared:

We are connecting patients with researchers, with funding, with biotech … The parent‐driven advocacy groups are the connectors. (A11)

### 3.4 Perceived benefits and barriers of interstakeholder collaboration throughout advanced neurotherapeutic development

Leaders perceived a degree of complexity regarding RDPO interactions with industry, other RDPOs, clinicians and researchers throughout advanced neurotherapeutic development.

#### 3.4.1 Benefits and barriers of RDPO‐industry collaboration: ‘A symbiotic relationship’

Among the cohort, 15/20 characterized RDPO‐industry collaborations as essential to facilitating advanced neurotherapeutic access. A fifth (4/20) of leaders suggested that the biopharmaceutical industry improved community research literacy through education (Table 2). According to 3/20 leaders, RDPO‐industry collaborations achieved a humanizing effect and promoted trust between stakeholders. One leader indicated that such partnerships helped personalize research from a pharmaceutical perspective, offering an emotional impetus for commercial goals. Others elaborated on this notion, suggesting that they instilled trust in patients and RDPO leaders, alike.

**Table 2:** RDPO perceptions of the benefits and challenges of collaboration amongst stakeholders throughout advanced neurotherapeutic development

Theme	Quote	Participant identification number
Perceived benefits of RDPO‐industry collaboration	Biggest benefit that I see to any of this is that we give people information. We give people facts. We take away the Santa Claus … and the wishful thinking and all of that, because we can give them data.	A10
	I really think that's an important connection to have, to help motivate [the biopharmaceutical industry] and help ground them and make sure that they continue to understand the purpose of what they're doing. It's not just a stem cell; they're actually people at the end of those stem cells that they're actually going to impact.	A11
Perceived barriers of RDPO‐industry collaboration	[The biopharmaceutical industry] will own the [intellectual property (IP)] … You won't have clear line of sight on the IP or on the research. You'll be a victim of whatever pricing comes in.	A15

Abbreviation: RDPO, rare disease patient organization.

Notably, 10/20 leaders raised the concept of collaborative lobbying for patient therapeutic access. To this teamwork, they ascribed particular importance, especially when describing rapid advances in regulatory approval, therapeutic development and product reimbursement, where robust RDPO‐industry relationships could serve facilitatory roles. Here, they voiced an appreciation for industry, suggesting that its involvement expedited these processes.

On the other hand, 11/20 of leaders expressed an awareness of bias and other barriers to effective collaboration, including industry's restriction of therapeutic access via high prices (2/10), with one expressing a sense of exploitation (Table 2).

#### 3.4.2 Benefits and barriers of inter‐RDPO collaboration: Passionate people, splinter groups and lots of personalities

Throughout this study, there appeared an emphasis on inter‐RDPO collaboration, with 18/20 RDPO leaders perceiving its importance to goal achievement, especially in research. According to more than half (11/20) of the cohort, mentorship and observational learning among RDPOs decreased duplication and increased efficiency. More than a third (7/20) of leaders perceived resource sharing as achievable, with 4/20 spontaneously proposing common office spaces and shared personnel. Given the multiplicity and low prevalence of distinct paediatric RNDs, RDPO leaders expressed a subtheme around the importance of collaboration in influencing health and research policy (Table 3).

**Table 3:** Illustrative quotes on perceived tension between RDPOs for therapeutic development, translation and support

Theme	Subtheme	Quote	Participant identification number
Perceived benefits of inter‐RDPO collaboration	Collaboration to influence health policy	There is a real risk that, when you talk about numbers in the rare disease space, no one gets heard because it's just lots and lots of little groups … there are a number of … umbrella organisations emerging that are really trying to harness the numbers.	A17
Perceived barriers of inter‐RDPO collaboration	Varying priorities	When I came into this space, I kind of figured everyone would want to work off the same page with the same endpoint. I wish it was that way. I find it very, very difficult to collaborate, to encourage collaborations … You have to sort of always be constantly thinking, ‘what is … in it for that person and why would they want to do that?’ And often, it's more than they would like to find a cure, which is unfortunate.	A1
	Focused cure‐seeking	There's a lot of families that come into this space and they want to get an answer for their child … And so, they become extremely focused on … getting researchers and whatever to be working on their child.	A9
	Different personalities	It depends [on] who's running that organisation … there's lots of personalities involved.	A12
	Rivalry	Just being more competitive than collaborative—so, wanting to own the space that they're working in and, yeah, not allowing … collaboration.	A6
	Competition for resources	I think everything should be properly funded, so that there is no fighting for money and funding … It is such a battle. It makes me quite sad that that is the case.	A19
	Logistical barriers	All of these groups are at varying stages of sophistication. So, some have much more developed systems and really strong relationships with all of their stakeholders, whereas some are quite new … We … need to be able to build the capacity of our patient organisations.	A3
	Overwhelming responsibilities	It's like drinking from a firehose sometimes. There's so much going on that you just can't … It's almost like you pick and choose your battles.	A18

Abbreviation: RDPO, rare disease patient organization.

Contrastingly, 13/20 leaders identified pervasive barriers to inter‐RDPO collaboration. Subthemes around varying priorities, focused cure‐seeking, different personalities, rivalry, competition for resources and logistical barriers dependent on organizational size, funding and sophistication emerged as sources of tension (Table 3). Additionally, RDPO leaders observed a subtheme around overwhelming responsibilities precluding effective collaboration, with some feeling compelled to prioritize immediate targets over collaborative efforts.

#### 3.4.3 The perceived need for improved interresearcher collaboration: Policies and bureaucracy hindering progress and harbouring frustrations

The cohort's quantitative surveys (18/19) affirmed the perceived importance of interresearcher data sharing throughout therapeutic development. Nevertheless, 11/20 leaders expressed frustrations around the field's current degree of data sharing. RDPO leaders commonly described an ‘ego drive’ (A5) among researchers that prevented data sharing. Competition, pride and career advancement were identified sources from which this inhibitive ‘ego drive’ derived, affecting all layers of the research hierarchy from researchers to universities and granting bodies:

I think it's a bit of an ego drive sometimes, you know. Who's going to write that first paper on that condition? Who's going to become the expert in that condition? (A5)

Three‐quarters (15/20) of leaders alluded to practical barriers of interresearcher collaboration. Of these leaders, two thirds (10/20) cited data management issues—namely, confidentiality, consent and ethics. A fifth (4/20) voiced concerns around the ‘reinvention of the wheel’ effectuated by inadequate data sharing. Even so, some (6/20) appreciated the role of privacy protocols in preventing data misuse. As one leader stated:

I think that's important, you know, if we didn't have those governance issues, then, you know, it's all open slather for everyone. (A7)

A majority (14/20) of RDPO leaders proposed solutions to overcome data‐sharing barriers and enable therapeutic development. Their recommendations ranged from promoting altruism in academia to mandating data sharing and improving financial and intellectual property protection. Half (10/20) described methods of coordinating collaboration and data sharing. From these leaders, most (6/20) noted current or previous attempts at implementing these strategies as RDPOs. For instance:

We are also building a network of researchers, so that there can be more collaboration and sharing of resources and knowledge. (A6)

Several (7/20) leaders commented on opportunistic lessons learned from the COVID‐19 pandemic, wherein teamwork and data‐sharing platforms overcame obstructive bureaucracy. This concept is exemplified by the quote:

Platforms were set up for COVID‐19 data sharing that I think have been quite successful, and I think we can learn a lot from that. (A6)

#### 3.4.4 Collaborations between RDPOs, clinicians and researchers: Consultations and partnerships

Whether maintaining patient contacts, lobbying for government funding or co‐designing research proposals, 14/20 RDPO leaders stated that they routinely engaged in RDPO‐clinician and RDPO‐researcher partnerships. Of these, 11/20 described consultation with researchers throughout therapeutic development. Collectively, researcher–clinician education and information distribution (6/20) also emerged as important RDPO responsibilities. Leaders credited researchers for improving community research and health literacy—whether through direct interactions with families or partnerships with RDPO leaders.

### 3.5 Perceived information needs regarding advanced neurotherapeutic development

At the heart of their advanced neurotherapeutic contributions and attitudes, leaders voiced an eagerness to remain well‐informed. They described a range of information practices, perceiving these as central to supporting community expectations and analysing advanced neurotherapeutic risks, benefits and uncertainties.

### 3.6 Information needs regarding advanced neurotherapeutic development in RDPO leaders and their communities

Most (17/20) leaders nominated clinician–researchers as their best information sources, especially around therapeutic innovation. In fact, 14/20 described active efforts to maintain familiarity and direct communication with clinicians and/or researchers. Leaders expressed an appreciation for personal contact, suggesting that direct interactions with experts invoked a sense of security in RDPOs and patients. As one stated:

Having someone speak to you directly always brings a sense of … personal connection and, like, safety … If you can chat with someone, and you know that person knows what they're talking about, then that's always going to feel better. (A20)

A majority (12/20) emphasized the need for more understandable resources on advanced neurotherapeutics:

When the drug company puts out the reports, they put out either the high science or the investor community information. There's nothing in the middle … so, that's one of the … issues that industry could really help us with is having that … in a more digestible form for the community. (A8)

They criticized the dearth of available resources for RDPOs and RND‐affected parents, whose baseline understandings were usually higher than the general public but lower than researchers. Almost half (8/20) of leaders identified the internet as a powerful, albeit ‘not always helpful’ (A2), resource. Specifically, they described social media as a potentially dangerous platform for the perpetuation of misleading parental anecdotes and false cures:

We have a Facebook page ….[which has] everything from the former US President's chlorine fixes for COVID…. through to, you name it, they've tried it. (A15)

Concurrently, the tension between secrecy and transparency appeared as a subtheme among 4/20 leaders. Ethics, governance and confidentiality issues emerged as perceived barriers to RDPO‐community information circulation around advanced neurotherapeutic trials:

[A biopharmaceutical company in the United States] may say, ‘oh yeah, we're bringing the trials to Australia’, and it's like, ‘well what's happening?’ And the community ask us, and we know, and we can't say anything. And it just seems like, why is this such a big secret? … We're not dealing … Weapons of Mass Destruction. (A8)

From the perspective of 9/20 leaders, neutrality was critical to thwarting misinformation and enabling informed consent among parents. Even so, several (5/20) RDPO leaders described feeling underinformed about advanced neurotherapeutics, attributing their knowledge gaps to being overstretched and service‐based. Another leader cautioned against the damaging potential of underinformed RDPOs:

Support groups and advocacy groups can be quite detrimental to clinical care because they can say and do the wrong thing and promote something… (A8)

To address informational demands surrounding advanced neurotherapeutics, 8/20 leaders raised the need for a central information resource. Leaders proposed the appointment of a credible, independent, intermediary organization, perhaps, an RDPO, to filter all advanced neurotherapeutic research opportunities into a website, fact sheet or other media.

**Figure 2:**
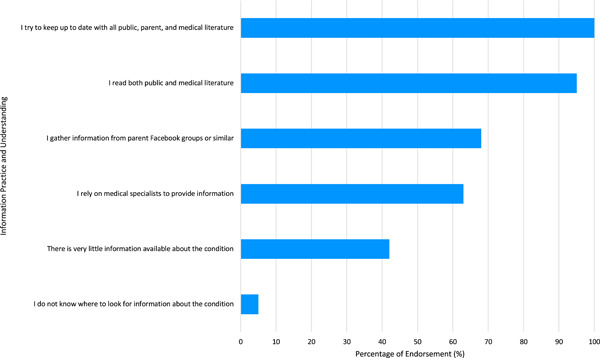
The information practices and understandings of advanced neurotherapeutics among RDPO leaders. Data were collected from 19 survey respondents. Endorsement included results with ‘agree and strongly agree’. RDPO, rare disease patient organization.

Quantitative survey data supplemented descriptions of RDPO information practices and understandings of advanced neurotherapeutics (Figure 2). The number of leaders demonstrating familiarity with specific treatments varied from 19/19 in gene therapies to only 5/19 in monoclonal antibody therapies. Information availability regarding advanced neurotherapeutics was considered ‘excellent’ by none, ‘very good’ by 3/19, ‘good’ by 10/19 and ‘poor’ by 2/19. Medical specialists (8/19) emerged as the most preferred information modality, followed by internet resources with frequently asked questions (6/19). Health professionals (13/19) were RDPO leaders' most trusted information sources, followed by disease‐specific websites (5/19).

### 3.7 Perceived psychosocial support needs associated with advanced neurotherapeutics

Leaders identified hope and desperation as community drivers for advanced neurotherapeutics, articulating their influence on parental expectations and risk‐benefit analyses. They recognized psychosocial supports as critical to empowering family‐centred decision‐making. There existed an overarching theme around insufficient psychosocial support for both RDPO leaders and the families that they served.

### 3.8 Hope, well‐being and desperation: The need for improved psychosocial support in RDPO leaders and patient families

Within the advanced neurotherapeutic realm, RDPO leaders perceived hope as a double‐edged sword. Notably, 12/20 RDPO leaders coupled hope with the potential to access advanced neurotherapeutics as part of one subtheme (Table 4). Contrastingly, RDPO leaders raised another subtheme around false hope, cautioning against fostering false hope in patient families and arguing that study ineligibility, adverse outcomes and failed advanced neurotherapeutic clinical trials could prove detrimental to mental health.

**Table 4:** RDPO perspectives and experiences of hope, well‐being and desperation across advanced neurotherapeutic development and translation in rare neurological diseases, as illustrated through quotes

Theme	Subtheme	Quote	Participant identification number
Hope	Hope around the potential to access advanced neurotherapeutics	Even just knowing that there's a clinical trial out there is actually useful for your mental health—just knowing that there's hope.	A11
	False hope associated with the potential to access advanced neurotherapeutics	I've seen lots of families quite devastated because … they've put all hope out into a research trial, but they haven't necessarily checked the eligibility criteria and things like that, and then realised later that their child's going to miss out.	A3
		You might get a parent's hopes up and then they can be dashed and then they can end up with huge mental health issues, because they're pinning everything on this clinical trial working, and it may not work.	A5
Well‐being	Advanced neurotherapeutic development impacting well‐being	I would say being part of a clinical trial can be emotionally exhausting. And I think that, obviously, that exhaustion and … those emotions would probably feel worse if then, on top of having to go through all that, then it doesn't work.	A20
	Uncertainty within RNDs and its impact on well‐being	The biggest thing that we live with in rare disease is uncertainty and it's everywhere, throughout all rare disease … It impacts on every part of living with a rare disease, of treating someone with a rare disease.	A16
		A huge number of carers are on antidepressants … when no one can give you an answer, I think that exacerbates the problem.	A5
		What does the process look like for the child? How … out of their normal environment ecosystem are they being placed? … Any kind of external stressors could … trigger a regression.	A18
Desperation	Informed consent versus passive listening	Some parents are just so desperate that they'll just try anything without even reading consent papers, just sign off, and then they're just so sleep deprived and desperate, they'll just do anything. And that might not necessarily be, you know, informed consent if they hadn't really understood—not just listened, but understood—the risks that may go with their clinical trial.	A5
		They're managing seizures and meltdowns, and … all of their day‐to‐day problems with these complex children … they just don't … [have] any cerebral space left for them to … really make such enormous decisions like … ‘will I, or won't I pursue, you know, my child to go into this clinical trial?’	A5
		The child did pass away from manageable symptoms, because the parents were so afraid that the child was going to be taken off the trial that they didn't report the side effects.	A8

Abbreviations: RDPO, rare disease patient organization; RND, rare neurological disease.

According to RDPO leaders, access to and involvement in advanced neurotherapeutic development could impact patient family well‐being, with negative repercussions around clinical trial inclusion materializing against the background of hope (Table 4). Fourteen out of 20 leaders explored the theme, well‐being, through the subtheme of navigating an inherently uncertain paediatric RND sphere, whose riskiness could provoke negative mental health outcomes in families. RDPO leaders also noted a sense of parental desperation, which could influence consent, decision‐making and outcome‐reporting processes throughout clinical trials (Table 4). Coupled with desperation, this field's baseline risk and uncertainty could allegedly fuel parents' willingness to trial therapies with ill‐defined safety‐benefit profiles for even the slightest prospect of cure:

I do think that they do have an appetite for risk, given the devastating nature of these conditions. (A6)

As an antidote to this, leaders emphasized RDPOs' crucial roles in providing individualized emotional support to patient families as they embarked on their own unique healthcare journeys through the evolving advanced neurotherapeutic landscape. A proportion of the cohort (2/20) described the need for improved RDPO‐delivered family support, especially around coping with new diagnoses. According to 5/20 leaders, a protracted diagnostic odyssey could subject families to immense emotional stress. Meanwhile, postnatal screening with the early diagnosis was perceived to invoke similar distress in unassuming parents with presymptomatic children.

[Family 1] couldn't handle that … [With rega to family 2], four years of thinking the worst and expecting the worst, to be told anything at that point, they would take it. But [family 1] were expecting and thinking they have a healthy child … there are different journeys. (A11)

RDPO representatives themselves expressed varied perspectives on how patient advocacy impacted their mental health. Some (3/20) associated their roles with high job satisfaction and moral duty. Most leaders reported DASS‐21 scores in the normal range for depression (16/17), anxiety (15/17) and stress (14/17) symptoms. However, 4/20 RDPO leaders, all of whom had at least one RND‐affected child, perceived an emotional toll associated with advocacy work.

**Figure 3:**
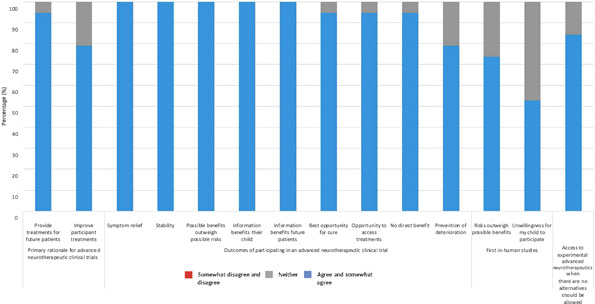
Opinions regarding the risks and benefits of advanced neurotherapeutics and advanced neurotherapeutic clinical trials. Data were collected from 19 survey respondents. Results were categorized as follows: ‘somewhat disagree and disagree’ = red; ‘neither agree nor disagree’ = grey and ‘agree and somewhat agree’ = blue.

Quantitative survey data supplemented narratives around determinants of advanced neurotherapeutic expectations, especially regarding applications, risks and benefits (Figure 3). RDPO leaders indicated that diseases with high mortality (18/19), severe symptoms (17/19), worsening progression (15/19) and/or no alternative therapies (15/19) deserved prioritization for experimental advanced neurotherapeutic interventions. Some also identified diseases either at early (11/19) or advanced (8/19) stages, but none indicated that diseases with existing alternative therapies warranted priority. Doctors experienced with advanced neurotherapeutics (10/19) emerged as leaders' most preferred modality for support, followed by RDPOs (5/19).

## 4 DISCUSSION

### 4.1 Thematic analysis

**Figure 4:**
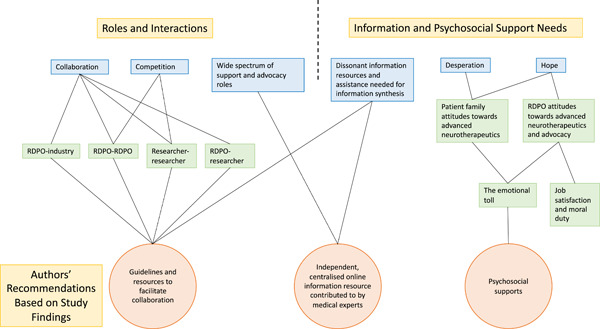
A thematic map outlining the relationships between RDPO roles, interactions, information and psychosocial support needs and recommendations for healthcare professionals involved in advanced neurotherapeutic development. Colour shadings are as follows: major themes = blue; most relevant subthemes = green; recommendations = orange. Themes and subthemes are denoted as rectangles. Recommendations are denoted as circles. RDPO, rare disease patient organization.

To our knowledge, this study is the first to comprehensively evaluate the perspectives of RDPO leaders around their roles, interactions, information requirements and psychosocial support needs in advanced neurotherapeutics. This study offers compelling insights into the integral role and complexity of RDPO involvement in this field. Its findings inform our own recommendations to medical professionals—namely, for the integration of patient voices throughout advanced neurotherapeutics (Figure 4).^8^, ^12^ Adopting these recommendations may improve clinical outcomes in a field where consumer engagement is increasingly present and valued.^44^

This study reaffirms a positive trend in patient research involvement observed throughout Western countries since the turn of the 21st century.^45^ While reinforcing previous literature on RDPO community and advocacy roles, it principally aligns with prior studies that have projected exponential growth in the influence of research‐engaged RDPOs.^12^ Consistent with studies from Australia, Europe and the United States, this present study captures the vastness of RDPO research commitments, which range from funding to data collection.^12^, ^34^, ^46^, ^47^ While earlier studies have characterized the counselling of research participants as an important, albeit less substantial, RDPO responsibility, nearly all (19/20) leaders in our study emphasized their active roles in preparing families for advanced neurotherapeutic clinical trials.^12^, ^48^ Our results may reflect organizational efforts to help patient families navigate an accelerative rise in advanced neurotherapeutic clinical trials.^47^ Interestingly, these findings appear alongside the subtheme of community empowerment. They align with studies depicting RDPOs as coalitions, whose research pursuits unify and empower their otherwise disenfranchized members.^49^

In our study, RDPO leaders unanimously described interstakeholder collaborations as pivotal in facilitating advanced neurotherapeutic development, corresponding with studies that have outlined the bidirectional relationship between consumer optimism and expanding public–private partnerships.^47^ Noting the heightened momentum within advanced neurotherapeutics, collaboration guidelines between research partners are required. This is particularly relevant to RDPO‐industry collaborations, which are historically associated with bias. Indeed, previous surveys in patient organizations have highlighted concerns around industry‐posed threats to objectivity, transparency, independence and public support.^8^, ^19^, ^50^ Recognizing RDPOs' growing significance to the industry, a 2018 study constructed collaboration guidelines based on mutual respect, accountability, commitment, transparency and trust.^51^

Notably, most studies investigating bias in RDPO‐industry collaborations have centred on research sponsorship, recruitment and outcome measure design.^8^, ^52^ Instead, leaders in our study ascribed importance to the humanizing aspects of collaboration. Their perceptions highlight the possibility for communication between industry and patients to relieve both parties of their arbitrarily ‘other’ statuses, thereby increasing patient receptivity, industry empathy and research efficiency. To acknowledge RDPOs' social growth, guidelines require revision, standardization and proper implementation.

Joint studies funded by RDPOs in comparable disease areas have previously been associated with economies of scale and increased likelihoods of identifying common drug targets and pathophysiological pathways.^18^, ^53^ While recognizing inter‐RDPO collaboration benefits, RDPO leaders acknowledged the coexistence of competition. They attributed group splintering to hitherto underexplored sources of tension, including resource availability, conflicting priorities and different personalities. Their classification of focused cure‐seeking as a barrier in RDPO leaders with RND‐affected children reinforces previous evidence suggesting that the overinvestment in cure observed among some advocacy groups could hinder research into other ancillary fields, including quality‐of‐life, social relationships and basic sciences.^8^, ^54^

Conforming with mixed methodology studies that have observed RDPO fragmentation issues before the advent of advanced neurotherapeutics, our cohort described a positive correlation between organizational sophistication and capacity for inter‐RDPO collaboration.^12^ At the national level, some umbrella organizations have conducted training programs to build the research capacity of small RDPOs.^23^, ^55^, ^56^ However, as per Australia's National Strategic Action Plan for Rare Diseases, resources facilitating meaningful RDPO research collaborations remain highly sought.^32^

While some RDPO leaders had continued to value the research enterprise over time, others had become disenchanted. Initially expecting researcher dedication to advanced neurotherapeutic discovery and improved patient outcomes, several leaders perceived challenges around inadequate data sharing, contributing to a feeling of disappointment. Notably, they criticized perceived vested interests among researchers and systemic flaws in the domain of academic career advancement. Their perspectives add to an academic reservoir of themes critiquing biomedical culture. Such themes include conflicts of interest, positive outcome bias, ‘reinvention of the wheel’ and insufficient harmonization of governance, intellectual property and research protocols.^8^, ^50^, ^57^, ^58^, ^59^, ^60^ Interestingly, their reflections on the COVID‐19 pandemic emphasize how a common sense of urgency among researchers can facilitate data‐sharing platforms and multinational, multicentre and multidisciplinary collaboration. Their perspectives reflect an optimism that a postpandemic culture of teamwork can mobilize researchers and expedite advanced neurotherapeutic development.^61^

Supporting their versatility within researcher partnerships, RDPO leaders extensively discussed their positions as research intermediaries, whose duties involved disseminating information and forging interresearcher connections. Prior studies have confirmed the effectiveness of RDPO‐researcher collaborations. Through co‐designed natural history recruitment‐retention strategies, one study achieved 97% retention across surviving participants, thus emphasizing the value of RDPOs in community educational outreach.^62^ This theme is underpinned by statute, with Australia's National Health and Medical Research Council Act 1992 valuing RDPO contributions through their mandate on meaningful consumer engagement throughout all research stages.^24^

While maintaining patient experiences at the heart of their professional expertise, RDPO leaders perceived their role to extend beyond the knowledge and skills of this traditional scope. We observed heightened rhetoric of empowerment relative to past studies. Though previous research has reported RDPO concerns around being patronized and financially exploited by researchers, this current study characterizes an active drive to appear as valued assets throughout advanced neurotherapeutic development.^8^, ^18^ In themselves, RDPO leaders perceived a readiness to gather current literature for clinician–researchers, thus highlighting a new engagement strategy founded on evidence rather than sympathy. This finding potentially delivers a solution to observations documented in previous studies that have alluded to a reluctance among RDPOs to mobilize researcher sympathy, due to fears of being deemed over‐emotional and unprofessional.^18^

While keeping informed of advanced neurotherapeutic literature, RDPOs continue to experience information access challenges, especially around health and social care.^10^, ^63^ This study found that familiarity with advanced neurotherapeutics varied greatly based on therapeutic type, with information availability deemed ‘excellent’ by none and ‘very good’ by only 3/19. Considering that RDPOs resoundingly struggle with accessing information from conferences and medical professionals, it is notable that, in this study, health specialists were nominated as the most preferred and trusted information modality.^63^ The value RDPO leaders attributed to direct communication with medical experts highlights the equal importance of information content and delivery. Even so, internet resources remain highly preferred and trusted information modalities.^63^ Like our own participants, prior mixed methodology studies in RDPOs have stressed difficulties around recognizing reliable online information, despite acknowledging the versatility of social media and other internet platforms.^10^, ^63^, ^64^, ^65^ Inconsistent information perpetuated by social media is especially relevant since individuals with rare disease diagnoses use social media platforms more frequently than the general population.^66^

International qualitative and mixed methodology studies demonstrate thematic consistency around an unmet need for centralized, reliable information hubs accessible to RDPOs.^10^, ^23^, ^64^ A study of rare disease patients, families, advocates and health professionals in Ireland argued that a locally relevant online information hub would best improve signposting of accredited information.^63^ Reiterating these themes within advanced neurotherapeutics, 40% of our cohort voiced their desire for a central information resource, preferably presided over by an independent, intermediary organization.

With many paediatric RNDs having complex, chronic phenotypes, psychosocial impacts on patient families are frequently pronounced. Nevertheless, RDPO leaders perceived that research opportunities involving advanced neurotherapeutics improved hope and resilience, enabling patient families to regain their sense of self‐determination.^8^ While acknowledging the importance of advanced neurotherapeutic studies, especially for conditions with increased mortality, symptomatic severity, prognosis and alternative treatment options, RDPO leaders cautioned against the psychosocial impact of participation in clinical trials on families. Although disappointment, false hope and uninformed consent have a long‐standing association with desperation throughout literature, research fatigue, as experienced by rare disease patients, is relatively underexplored.^8^, ^67^, ^68^ Our study supports the standardization and centralization of data repositories to reduce familial burden. Moreover, it reveals the need to assist RDPOs in supporting families psychosocially throughout advanced neurotherapeutic studies.

Interestingly, the unmet need for emotional support extends beyond patient families to RDPO leaders, themselves. This result aligns with previous evidence highlighting the significant proportion of RDPOs founded by rare disease‐affected parents and families.^12^ Although prior studies framed the research involvement of personally impacted RDPO leaders as a coping mechanism, this current study introduces a new concern—that is, the emotional toll of RND advocacy, itself.^8^ Notably, Australia's National Strategic Action Plan for Rare Diseases recommends increased mental health education for RDPO leaders and their communities.^32^ However, improved access to psychologists and other psychosocial supports may be needed.

### 4.2 Strengths and limitations

This study's convergent mixed methodology design integrated standardized, generalizable, quantitative data with rich, subjective perspectives, allowing the complementation of interpretivist and positivist paradigms.^69^ In doing so, this methodological triangulation capitalizes on data reflecting lived experiences and participant‐identified priorities.^69^, ^70^ Utilizing a standardized guide and facilitator throughout interviews minimized wording and loaded question biases. Member checking—the active solicitation of participant feedback on data interpretational accuracy—further enhanced our study's credibility. This study acknowledged the influence of researcher preconceptions on data collection, analysis and interpretation, incorporating weekly multidisciplinary team progress meetings into its methodology (Supporting Information: Appendix A). Investigator triangulation involving eight researchers and two coders throughout coding tree construction and coding respectively added breadth to findings. Moreover, the high intercoder reliability rate (97%) conformed to qualitative research standards. Complying with well‐established quality and trustworthiness criteria, these strategies increased the study's methodological rigour.^71^

Nevertheless, there are limitations to be considered when interpreting our results. Although we achieved code saturation (no new themes) at 10 transcripts and meaning saturation (no new perspectives) at 20 transcripts, we may have captured greater attitudinal diversity with a larger sample size.^72^ Indeed, only 3/20 participants represented RDPOs based outside Australia, limiting the transferability of themes across global healthcare systems. Nonetheless, we believe that our sample's perspectives are still highly relevant to the RDPO space, considering our comprehensive sampling technique and participant heterogeneity. In fact, the sample size enabled our focus on data quality, allowing us to characterize richly textured insights or ‘thick’ data.^72^, ^73^ Though population sampling would have minimized selection bias and maximized generalizability, purposive sampling supported the depth of our analysis by identifying highly relevant cases.^73^

### 4.3 Recommendations and future directions

This study has several implications for future advanced neurotherapeutic development. The perceived strengths among RDPOs in patient advocacy, information dissemination and social engagement should be harnessed to better empower patients and appreciate their experiences around RND and treatment. With advanced neurotherapeutics rapidly evolving, the need for collaboration guidelines between RDPOs, industry and researchers is increasingly apparent. Early RDPO engagement in the co‐design of advanced neurotherapeutic trials and studies appears essential to developing pathways that strengthen interstakeholder partnerships, optimize research outcomes, support patient families throughout their healthcare journeys and promote equitable, transparent access to novel interventions.

From the perspective of RDPOs, whose most preferred information modality remains medical experts, improved information delivery and standardized resources appear essential. A centralized online information resource is necessary to assist RDPOs in identifying and synthesizing reliable advanced neurotherapeutic information. Improved distributive information media for RDPOs would not only equip RDPO leaders for constructive interstakeholder discourse but also, enhance the quality of scientific information relayed to patient communities. Furthermore, our study captures the unmet mental health needs of both RDPO leaders and their communities. Healthcare systems require psychosocial support sensitive to the unique challenges of these stakeholders. Based on our findings, future pilot studies could co‐design and evaluate the effectiveness of such resources for implementation.

## 5 CONCLUSION

The consumer's voice is increasingly prominent across advanced neurotherapeutic development. This study reveals challenges faced by RDPOs around lacking collaboration guidelines, dissonant information access and mental health concerns. However, it also captures the valuable contributions of RDPOs to biomedical progress. With inequities still ubiquitous throughout the RND sector, there is a compelling demand for collaboration and research to address a serious unmet need. Further initiatives are required to inform and empower RDPOs as they advocate for patient families in this inherently passionate space.

## AUTHOR CONTRIBUTIONS

Christina Q. Nguyen played a prominent role in the project's conception. She was the interviewer for interviews and the primary coder during data analysis. She synthesized all qualitative and quantitative data. She composed the first manuscript draft and made major revisions to all subsequent publication drafts. Didu Kariyawasam played a prominent role in the project's conception. She was the secondary coder during data analysis. She contributed to major revisions of the final publication draft. Kristine Alba‐Concepcion was the facilitator of interviews and senior clinical research assistant, who addressed logistical concerns throughout the project. She reviewed interview transcripts and participated in coding review meetings during data analysis. She contributed to major revisions of the final publication draft. Sarah Grattan was a research assistant who addressed logistical concerns throughout the project. She reviewed interview transcripts and participated in coding review meetings during data analysis. She contributed to major revisions of the final publication draft. Kate Hetherington played a prominent role in the project's conception. She reviewed interview transcripts and participated in coding review meetings during data analysis. She contributed to major revisions of the final publication draft. Claire E. Wakefield played a prominent role in the project's conception. She reviewed interview transcripts and participated in coding review meetings during data analysis. She contributed to major revisions of the final publication draft. Susan Woolfenden played a prominent role in the project's conception. She contributed to major revisions of the final publication draft. Russell C. Dale played a prominent role in the project's conception. He contributed to major revisions of the final publication draft. Elizabeth E. Palmer played a prominent role in the project's conception. She was a co‐supervisor of this project. She reviewed interview transcripts and participated in coding review meetings during data analysis. She contributed to major revisions of the final publication draft. Michelle A. Farrar played a prominent role in the project's conception. She was the primary supervisor of this project. She reviewed interview transcripts and participated in coding review meetings during data analysis. She contributed to major revisions of the final publication draft.

## CONFLICTS OF INTEREST

K. A.‐C. receives support from the Ainsworth Foundation. K. H. receives support from Luminesce Alliance and the Zero Childhood Cancer National Personalised Medicine Program. C. E. W. receives support from the National Health and Medical Research Council (APP2008300). S. W. receives support from National Health and Medical Research Council (1158954). The remaining authors declare no conflict of interest.

## ETHICS STATEMENT

The study was approved by the Sydney Children's Hospital Network Human Research Ethics Committee (2020/ETH02748).

## Supporting information

Supporting information.

Supporting information.

Supporting information.

Supporting information.

Supporting information.

## Data Availability

Data that underlie the results reported in this article may be available on request, after de‐identification. Applicants willing to receive the data should apply between 1 and 12 months after the manuscript has been published and should demonstrate that the proposed use of the data has been approved by an independent review committee identified for this purpose. The data request should be sent to the corresponding author, M. A. F.

## References

[1] Richter T , Nestler‐Parr S , Babela R , et al. Rare disease terminology and definitions—a systematic global review: report of the ISPOR rare disease special interest group. Value Health. 2015;18(6):906‐914. 26409619 10.1016/j.jval.2015.05.008

[2] Nguengang Wakap S , Lambert DM , Olry A , et al. Estimating cumulative point prevalence of rare diseases: analysis of the orphanet database. Eur J Hum Genet. 2020;28(2):165‐173. 31527858 10.1038/s41431-019-0508-0 PMC6974615

[3] The Lancet Neurology . Rare advances for rare diseases. Lancet Neurol. 2016;16(1):1. 10.1016/S1474-4422(16)30352-0 27979337

[4] Walker CE , Mahede T , Davis G , et al. The collective impact of rare diseases in Western Australia: an estimate using a population‐based cohort. Genet Med. 2017;19(5):546‐552. 27657686 10.1038/gim.2016.143 PMC5440569

[5] Peiris V , Xu K , Agler HL , et al. Children and adults with rare diseases need innovative medical devices. J Med Device. 2018;12(3):347011‐347018. 10.1115/1.4040489 PMC6123063 30397422

[6] United States Food and Drug Administration: Center for Drug Evaluation and Research Office of New Drugs . Roadmap to patient‐focused outcome measurement in clinical trials. 2015. Accessed April 6, 2021. https://www.fda.gov/media/87004/download

[7] Depping MK , Uhlenbusch N , von Kodolitsch Y , Klose HFE , Mautner V‐F , Löwe B . Supportive care needs of patients with rare chronic diseases: multi‐method, cross‐sectional study. Orphanet J Rare Dis. 2021;16(1):44. 33482869 10.1186/s13023-020-01660-w PMC7825171

[8] Pinto D , Martin D , Chenhall R . Chasing cures: rewards and risks for rare disease patient organisations involved in research. Biosocieties. 2018;13(1):123‐147.

[9] Forsythe LP , Szydlowski V , Murad MH , et al. A systematic review of approaches for engaging patients for research on rare diseases. J Gen Intern Med. 2014;29(suppl 3):788‐800. 25047393 10.1007/s11606-014-2895-9 PMC4124116

[10] McMullan J , Crowe AL , Bailie C , et al. Improvements needed to support people living and working with a rare disease in Northern Ireland: current rare disease support perceived as inadequate. Orphanet J Rare Dis. 2020;15(1):315. 33168042 10.1186/s13023-020-01559-6 PMC7649905

[11] Black AP , Baker M . The impact of parent advocacy groups, the internet, and social networking on rare diseases: the IDEA League and IDEA League United Kingdom example. Epilepsia. 2011;52:102‐104. 21463291 10.1111/j.1528-1167.2011.03013.x

[12] Pinto D , Martin D , Chenhall R . The involvement of patient organisations in rare disease research: a mixed methods study in Australia. Orphanet J Rare Dis. 2016;11(1):2. 26754025 10.1186/s13023-016-0382-6 PMC4709899

[13] Gómez‐Zúñiga B , Pulido Moyano R , Pousada Fernández M , García Oliva A , Armayones Ruiz M . The experience of parents of children with rare diseases when communicating with healthcare professionals: towards an integrative theory of trust. Orphanet J Rare Dis. 2019;14(1):159. 31253163 10.1186/s13023-019-1134-1 PMC6599337

[14] McCormack P , Kole A , Gainotti S , et al. “You should at least ask”. the expectations, hopes and fears of rare disease patients on large‐scale data and biomaterial sharing for genomics research. Eur J Hum Genet. 2016;24(10):1403‐1408. 27049302 10.1038/ejhg.2016.30 PMC5027679

[15] Mayhew A , Mazzone ES , Eagle M , et al. Development of the performance of the upper limb module for Duchenne muscular dystrophy. Dev Med Child Neurol. 2013;55(11):1038‐1045. 23902233 10.1111/dmcn.12213

[16] Band H , Stehr F , Murphy N . Contribution of patient organisations to the NCLs. Biochim Biophys Acta Mol Basis Dis. 2020;1866(9):165773. 32224155 10.1016/j.bbadis.2020.165773

[17] Tones M , Cross M , Simons C , et al. Research protocol: the initiation, design and establishment of the Global Angelman Syndrome Registry. J Intellect Disabil Res. 2018;62(5):431‐443. 29633452 10.1111/jir.12482

[18] Panofsky A . Generating sociability to drive science: patient advocacy organizations and genetics research. Soc Stud Sci. 2011;41(1):31‐57. 21553639 10.1177/0306312710385852

[19] Koay PP , Sharp RR . The role of patient advocacy organizations in shaping genomic science. Annu Rev Genomics Hum Genet. 2013;14(1):579‐595. 23875802 10.1146/annurev-genom-091212-153525

[20] Privolizzi R , Chu WS , Tijani M , Ng J . Viral gene therapy for paediatric neurological diseases: progress to clinical reality. Dev Med Child Neurol. 2021;63(9):1019‐1029. 33834479 10.1111/dmcn.14885

[21] Tambuyzer E , Vandendriessche B , Austin CP , et al. Therapies for rare diseases: therapeutic modalities, progress and challenges ahead. Nat Rev Drug Discov. 2020;19(2):93‐111. 31836861 10.1038/s41573-019-0049-9

[22] Sarpatwari A , Beall RF , Abdurrob A , He M , Kesselheim AS . Evaluating the impact of the Orphan Drug Act's seven‐year market exclusivity period. Health Aff (Millwood). 2018;37(5):732‐737. 29733729 10.1377/hlthaff.2017.1179

[23] Choudhury MC , Saberwal G . The role of patient organizations in the rare disease ecosystem in India: an interview based study. Orphanet J Rare Dis. 2019;14(1):117. 31142331 10.1186/s13023-019-1093-6 PMC6542017

[24] National Health and Medical Research Council . Ideas Grants Peer Review Guidelines. Canberra National Health and Medical Research Council; 2021.

[25] National Health and Medical Research Council . Consumer and Community Engagement. National Health and Medical Research Council; 2020. Accessed March 16, 2021. https://www.nhmrc.gov.au/about-us/consumer-and-community-engagement

[26] National Health and Medical Research Council, Consumers Health Forum of Australia . Statement on Consumer and Community Involvement in Health and Medical Research. National Health and Medical Research Council, Consumers Health Forum of Australia; 2016.

[27] Farrar MA , Kiernan MC . Spinal muscular atrophy—the dawning of a new era. Nat Rev Neurol. 2020;16(11):593‐594. 32978515 10.1038/s41582-020-00410-7

[28] Australian Government Department of Health . Guidelines for the Treatment of Late‐infantile Onset Batten Disease Through the Life Saving Drugs Program. Australian Government Department of Health; 2019.

[29] Farrar MA , Carey KA , Paguinto S‐G , Kasparian NA , De Abreu Lourenço R . “The whole game is changing and you've got hope”: Australian perspectives on treatment decision making in spinal muscular atrophy. Patient. 2020;13(4):389‐400. 32266662 10.1007/s40271-020-00415-w

[30] Kariyawasam D , Alexander IE , Kurian M , Farrar MA . Great expectations: virus‐mediated gene therapy in neurological disorders. J Neurol Neurosurg Psychiatry. 2020;91(8):849‐860. 32503884 10.1136/jnnp-2019-322327

[31] Raffai F , Timmis O . Building the patient community. Gene Ther. 2017;24(9):547‐550. 28467403 10.1038/gt.2017.33

[32] Australian Government Department of Health . The National Strategic Action Plan for Rare Diseases. Canberra Australian Government Department of Health; 2020.

[33] Nori M , Fisher‐Vance D , Wuerth L , Colenso R , Donovan D . The global role of patients, advocates and caregivers in rare diseases. Future Rare Dis. 2022;2:1‐14.

[34] Landy DC , Brinich MA , Colten ME , Horn EJ , Terry SF , Sharp RR . How disease advocacy organizations participate in clinical research: a survey of genetic organizations. Genet Med. 2012;14(2):223‐228. 22261756 10.1038/gim.0b013e3182310ba0

[35] Augustine EF , Adams HR , Mink JW . Clinical trials in rare disease: challenges and opportunities. J Child Neurol. 2013;28(9):1142‐1150. 24014509 10.1177/0883073813495959 PMC3964003

[36] Iglesias‐Lopez C , Agustí A , Obach M , Vallano A . Regulatory framework for advanced therapy medicinal products in Europe and United States. Front Pharmacol. 2019;10(921):2711‐2716. 10.3389/fphar.2019.00921 PMC6728416 31543814

[37] Dufosset M , Tosello B , Le Coz P , Chabrol B . New ethical challenges in the management of rare pediatric diseases with innovative therapies. Arch Pediatr. 2021;28(4):311‐318. 33814267 10.1016/j.arcped.2021.02.004

[38] Nguyen CQ , Alba‐Concepcion K , Palmer EE , Scully JL , Millis N , Farrar MA . The involvement of rare disease patient organisations in therapeutic innovation across rare paediatric neurological conditions: a narrative review. Orphanet J Rare Dis. 2022;17(1):167. 35436886 10.1186/s13023-022-02317-6 PMC9014615

[39] Rare Voices Australia (RVA) . Rare Voices Australia Partner Organisations. 2021. Accessed June 20, 2021. https://rarevoices.org.au/a-z-of-partners

[40] Lovibond SH , Lovibond PF . Manual for the Depression Anxiety Stress Scales. Psychology Foundation of Australia; 1995.

[41] Fetters MD , Curry LA , Creswell JW . Achieving integration in mixed methods designs‐principles and practices. Health Serv Res. 2013;48(6 pt2):2134‐2156. 24279835 10.1111/1475-6773.12117 PMC4097839

[42] Johnson RB , Onwuegbuzie AJ , Turner LA . Toward a definition of mixed methods research. J Mix Methods Res. 2007;1(2):112‐133.

[43] Miles MB , Huberman AM , Saldaña J . Qualitative Data Analysis: A Methods Sourcebook. 3rd ed. SAGE Publications; 2014.

[44] Morel T , Aymé S , Cassiman D , Simoens S , Morgan M , Vandebroek M . Quantifying benefit‐risk preferences for new medicines in rare disease patients and caregivers. Orphanet J Rare Dis. 2016;11(1):70. 27225337 10.1186/s13023-016-0444-9 PMC4881055

[45] Domecq JP , Prutsky G , Elraiyah T , et al. Patient engagement in research: a systematic review. BMC Health Serv Res. 2014;14:89. 24568690 10.1186/1472-6963-14-89 PMC3938901

[46] Tejada P . EURORDIS Survey on “European Rare Disease Patient Groups in Research: Current Role and Priorities for the Future”. EURORDIS: Rare diseases of Europe; 2016. Accessed November 6, 2021. https://www.eurordis.org/content/survey-patient-groups-research

[47] Groft S , De la Paz M , Taruscio D . Progress, challenges, and global approaches to rare diseases. Acta Paediatr. 2021;110:2711‐2716. 34105798 10.1111/apa.15974

[48] Mavris M , Le Cam Y . Involvement of patient organisations in research and development of orphan drugs for rare diseases in Europe. Mol Syndromol. 2012;3(5):237‐243. 23293582 10.1159/000342758 PMC3531929

[49] Zhu X , Smith R , Parrott R . Living with a rare health condition: the influence of a support community and public stigma on communication, stress, and available support. J Appl Commun Res. 2017;45:1‐20. 10.1080/00909882.2017.1288292 PMC5793934 29398734

[50] Lexchin J . Sponsorship bias in clinical research. Int J Risk Saf Med. 2012;24(4):233‐242. 23135338 10.3233/JRS-2012-0574

[51] Stein S , Bogard E , Boice N , et al. Principles for interactions with biopharmaceutical companies: the development of guidelines for patient advocacy organizations in the field of rare diseases. Orphanet J Rare Dis. 2018;13(1):18. 29357903 10.1186/s13023-018-0761-2 PMC5778794

[52] Gaasterland CMW , van der Weide MCJ , du Prie‐Olthof MJ , et al. The patient's view on rare disease trial design—a qualitative study. Orphanet J Rare Dis. 2019;14(1):31. 30732630 10.1186/s13023-019-1002-z PMC6367834

[53] Litterman NK , Rhee M , Swinney DC , Ekins S . Collaboration for rare disease drug discovery research. F1000Res. 2014;3:261. 25685324 10.12688/f1000research.5564.1 PMC4314660

[54] Dresser R . When Science Offers Salvation: Patient Advocacy and Research Ethics. Oxford University Press; 2001.

[55] Lacaze P , Millis N , Fookes M , et al. Rare disease registries: a call to action. Intern Med J. 2017;47(9):1075‐1079. 28891182 10.1111/imj.13528

[56] Cismondi IA , Kohan R , Adams H , et al. Guidelines for incorporating scientific knowledge and practice on rare diseases into higher education: neuronal ceroid lipofuscinoses as a model disorder. Biochim Biophys Acta Mol Basis Dis. 2015;1852(10, part B):2316‐2323. 10.1016/j.bbadis.2015.06.018 26117801

[57] Morris ZS , Wooding S , Grant J . The answer is 17 years, what is the question: understanding time lags in translational research. J R Soc Med. 2011;104(12):510‐520. 22179294 10.1258/jrsm.2011.110180 PMC3241518

[58] Fanelli D . Negative results are disappearing from most disciplines and countries. Scientometrics. 2012;90(3):891‐904.

[59] Austin MA , Hair MS , Fullerton SM . Research guidelines in the era of large‐scale collaborations: an analysis of genome‐wide association study consortia. Am J Epidemiol. 2012;175(9):962‐969. 22491085 10.1093/aje/kwr441 PMC3339312

[60] Goldacre B . Bad Pharma: How Drug Companies Mislead Doctors and Harm Patients. HarperCollins Publishers; 2013.

[61] Chung CCY , Ng YNC , Jain R , Chung BHY . A thematic study: impact of COVID‐19 pandemic on rare disease organisations and patients across ten jurisdictions in the Asia Pacific region. Orphanet J Rare Dis. 2021;16(1):119. 33673852 10.1186/s13023-021-01766-9 PMC7935006

[62] Bartlett A , Kolb SJ , Kingsley A , et al. Recruitment & retention program for the NeuroNEXT SMA biomarker study: super babies for SMA. Contemp Clin Trials Commun. 2018;11:113‐119. 30094386 10.1016/j.conctc.2018.07.002 PMC6072892

[63] Crowe AL , McKnight AJ , McAneney H . Communication needs for individuals with rare diseases within and around the healthcare system of Northern Ireland. Front Public Health. 2019;7(236):236. 31497589 10.3389/fpubh.2019.00236 PMC6712370

[64] Li X , Lu Z , Zhang J , et al. The urgent need to empower rare disease organizations in China: an interview‐based study. Orphanet J Rare Dis. 2020;15(1):282. 33046132 10.1186/s13023-020-01568-5 PMC7552513

[65] Pauer F , Litzkendorf S , Göbel J , Storf H , Zeidler J , Graf von der Schulenburg J‐M . Rare diseases on the internet: an assessment of the quality of online information. J Med Internet Res. 2017;19:e23. 28100442 10.2196/jmir.7056 PMC5288561

[66] Rocha HM , Savatt JM , Riggs ER , Wagner JK , Faucett WA , Martin CL . Incorporating social media into your support tool box: points to consider from genetics‐based communities. J Genet Couns. 2018;27(2):470‐480. 29130143 10.1007/s10897-017-0170-z PMC8340936

[67] Eijkholt M . Medicine's collision with false hope: the False Hope Harms (FHH) argument. Bioethics. 2020;34(7):703‐711. 32134519 10.1111/bioe.12731 PMC7664828

[68] Babac A , von Friedrichs V , Litzkendorf S , Zeidler J , Damm K , Graf von der Schulenburg JM . Integrating patient perspectives in medical decision‐making: a qualitative interview study examining potentials within the rare disease information exchange process in practice. BMC Med Inform Decis Mak. 2019;19(1):188. 31533712 10.1186/s12911-019-0911-z PMC6751820

[69] Regnault A , Willgoss T , Barbic S , International Society for Quality of Life Research Mixed Methods Special Interest Group . Towards the use of mixed methods inquiry as best practice in health outcomes research. J Patient Rep Outcomes. 2017;2(1):19. 29757311 10.1186/s41687-018-0043-8 PMC5934918

[70] Rahman MS . The advantages and disadvantages of using qualitative and quantitative approaches and methods in language “testing and assessment” research: a literature review. J Educ Learn. 2016;6:102.

[71] Korstjens I , Moser A . Series: practical guidance to qualitative research. Part 4: trustworthiness and publishing. Eur J Gen Pract. 2018;24(1):120‐124. 29202616 10.1080/13814788.2017.1375092 PMC8816392

[72] Hennink MM , Kaiser BN , Marconi VC . Code saturation versus meaning saturation: how many interviews are enough? Qual Health Res. 2016;27(4):591‐608. 27670770 10.1177/1049732316665344 PMC9359070

[73] Vasileiou K , Barnett J , Thorpe S , Young T . Characterising and justifying sample size sufficiency in interview‐based studies: systematic analysis of qualitative health research over a 15‐year period. BMC Med Res Methodol. 2018;18:148. 30463515 10.1186/s12874-018-0594-7 PMC6249736

