# Supplementary material for: ‘Advocacy groups are the connectors’: Experiences and contributions of rare disease patient organization leaders in advanced neurotherapeutics
Source: Health Expect. 2022 Oct 28;25(6):3175–91. doi: 10.1111/hex.13625 (PMC9700154; doi:10.1111/hex.13625)
Supplement: Supplementary file 1 — Supporting information. [file HEX-25--s002.docx]

**Appendix A**: Table of researcher characteristics

| Researcher (credentials) | Occupation at time of study | Gender | Experience and training | Relationship to participants |
| --- | --- | --- | --- | --- |
| CQN | Medical student | Female | 5 years of medical education, and fundamental training in NVivo 12 Pro | No prior relationship with participants. Interviewer of semi-structured interviews. |
| DK (BA, MA (Cantab), MBBS, MRCPCH, CCST (UK), FRACP) | Paediatric neurologist | Female | >10 years of experience in research and paediatric neurology | Prior interactions with some participants due to clinical role. Did not attend or participate in semi-structured interviews. |
| KA-C (BSc, MD, MPH, MHS) | Senior clinical research officer | Female | >10 years of experience in clinical research | No prior relationship to participants. Facilitator  of semi-structured interviews. |
| SG (BMedSc) | Research assistant | Female | 4.5 years of experience in research | No prior relationship with participants. |
| KH (BSc (Psych) (Hons), MPsych (Clinical), PhD) | Postdoctoral research fellow, clinical psychologist | Female | >10 years of experience in clinical practice and research | No prior relationship with participants. |
| CEW (BPsych (Hons1), MPH, PhD) | Professor, medical psychologist | Female | >10 years of experience in research | No prior relationship with participants. |
| SW (MBBS, PhD) | Associate professor, paediatrician | Female | >10 years of experience in research | No prior relationship with participants. |
| RCD (MRCP, PhD) | Professor, paediatric neurologist | Male | >10 years of experience in research and paediatric neurology | Prior interactions with some participants due to clinical role. Did not attend or participate in semi-structured interviews. |
| EEP (MBBS, PhD) | Clinical geneticist | Female | >10 years of experience in research and clinical genetics | Prior interactions with some participants due to clinical role. Did not attend or participate in semi-structured interviews. |
| MAF (MBBS (Hons), FRACP, PhD) | Associate professor, paediatric neurologist | Female | >10 years of experience in  research and  paediatric neurology | Prior interactions with some participants due to clinical role. Did not attend or participate in semi-structured interviews. |
