# Supplementary material for: ‘Advocacy groups are the connectors’: Experiences and contributions of rare disease patient organization leaders in advanced neurotherapeutics
Source: Health Expect. 2022 Oct 28;25(6):3175–91. doi: 10.1111/hex.13625 (PMC9700154; doi:10.1111/hex.13625)
Supplement: Supplementary file 2 — Supporting information. [file HEX-25--s001.docx]

**Appendix B**: Interview discussion guide (developed in conjunction with the Sydney Children’s Hospitals Network research team)

***Introduction***

- Facilitator thanks participant for their time and introduces interviewer.
- Interviewer introduces the discussion:

Hello .

Thank you so much for taking the time to conduct this interview with us today. This interview centres on the roles, interactions, attitudes, and awareness of rare disease patient organisations in advanced neurotherapeutic development.

Are you happy for this conversation to be recorded?

Throughout this interview, we will be discussing a number of topics. Everything you say will be reported as anonymous. Please feel free to provide as much information as you like, and to stop me at any time, if you need a break.

***Questions***

1. As a member of , can you please tell me about your experiences advocating for people with ?
2. When you think about potential treatments for , what comes to mind?
3. What roles do your organisation play with regards to the development of new, advanced, and emerging therapies?
4. If you were to provide information and education to families about a new therapy, what would you want to know about that therapy first?
5. How would you like to find out information about a certain therapy?
6. What is your understanding of the processes by which experimental therapies (such as gene therapy or stem cell therapy) are tested in humans?
7. After experimental clinical trials are completed, it is typical that patients are followed up over time to see the long-term effects of the treatment. What is your view on long-term follow-up after an experimental intervention?
8. What are your views on collaborations between patient advocates and biopharmaceutical companies?
9. What is your opinion on the importance of collaborating with other rare disease patient organisations, especially in research?
10. The data collected during clinical trials is sometimes available for other scientists to view and use. In other trials, the data is not made available. What is your view on the sharing of data collected in clinical trials?
11. Which types of diseases do you think should be selected for new experimental interventions with substantial uncertainties surrounding the risks and benefits?
12. What do you think would be helpful to share with families whose child has just been diagnosed with ?
13. Is there anything else you would like to know?

***Conclusion***

Thank you so much for your time today. I just wanted to ask whether our facilitator has anything also to add before we finish up?
