# Supplementary material for: ‘Advocacy groups are the connectors’: Experiences and contributions of rare disease patient organization leaders in advanced neurotherapeutics
Source: Health Expect. 2022 Oct 28;25(6):3175–91. doi: 10.1111/hex.13625 (PMC9700154; doi:10.1111/hex.13625)
Supplement: Supplementary file 3 — Supporting information. [file HEX-25--s003.docx]

**Appendix C**: Questionnaire 1 (developed in conjunction with the Sydney Children’s Hospitals Network research team)

***Section 1: Personal details***

1. What is your date of birth?
2. What is your gender?

| □ Male | □ Female | □ Other |
| --- | --- | --- |

1. What is the highest level of education you have completed?

| □ Year 10 or below | □ Apprenticeship | □ University Degree |
| --- | --- | --- |
| □ Year 12 | □ Certificate/Diploma | □ Post-graduate Degree |

1. What is your current employment situation?

| **Employed** |  |  |
| --- | --- | --- |
| □ Full time | □ Part time | □ Casual |

| **Not Employed** |  |  |
| --- | --- | --- |
| □ Actively seeking work | □ Not seeking work | □ Retired |
| □ Student | □ Home duties |  |

1. What is your current marital status?

| □ Never married/never de facto | □ Currently married or de facto |
| --- | --- |
| □ Separated/Divorced | □ Widowed |

1. What is your cultural background? (e.g. Australian, Chinese, Greek)

| □ Yes | □ No |
| --- | --- |

1. Do you identify as having an Aboriginal or Torres Strait Islander background?
2. What is your first language?
3. What is your religion?

| □ No religion | □ Buddhism | □ Christianity |
| --- | --- | --- |
| □ Islam | □ Hinduism | □ Judaism |
| □ Sikhism | □ Other | |

| □ Yes | □ No |
| --- | --- |

1. Do you have any children?

If yes, please fill in the following table for each child.

| **Child** | | | | | |
| --- | --- | --- | --- | --- | --- |
| Gender: | □ Male | □ Female | | □ Other | |
| Date of Birth: |  | | | | |
| Diagnosed with a neurological condition? | □ Yes | □ No | | If yes, complete below | |
| Name of neurological condition |  | | | | |
| Age at diagnosis |  | |  | |  |

| □ Yes | □ No |
| --- | --- |

1. Do you belong to a patient advocacy or support organisation?
2. If yes, what disease or group of diseases does the organisation represent?
3. What is your role in the organisation?

| □ General member | □ Board member | □ Chief executive officer |
| --- | --- | --- |
| □ Administrative assistant | □ Secretary | □ Board member |
| □ Other | | |

1. Are you employed by the organisation?

| □ Yes | □ No |
| --- | --- |

1. If yes, are you:

| □ Full time | □ Part time | □ Casual |
| --- | --- | --- |
| □ Volunteer |  |  |

1. Please rate how much you agree with the following statements regarding information about the condition your organisation represents?

|  | Strongly disagree | Disagree | Neither agree nor disagree | Agree | Strongly Agree |
| --- | --- | --- | --- | --- | --- |
| I try to keep up to date will all public, parent and medical literature | □ | □ | □ | □ | □ |
| I read both public and medical literature | □ | □ | □ | □ | □ |
| I gather information from parent Facebook groups or similar | □ | □ | □ | □ | □ |
| I rely on medical specialists to provide information | □ | □ | □ | □ | □ |
| There is very little information available about the condition | □ | □ | □ | □ | □ |
| I do not know where to look for information about the condition | □ | □ | □ | □ | □ |

***Section 2: Personal understanding of advanced therapies for neurological disorders***

1. In general, how familiar are you about the following advanced therapies?

|  | Not at all | Not very | Neither | Somewhat | Very |
| --- | --- | --- | --- | --- | --- |
| Gene therapies | □ | □ | □ | □ | □ |
| Stem cell therapies | □ | □ | □ | □ | □ |
| Monoclonal antibody therapies | □ | □ | □ | □ | □ |
| Neurostimulators (deep brain stimulation) | □ | □ | □ | □ | □ |

1. Please tick the answer that best reflects your thoughts about the following statements.

|  | Disagree | Somewhat disagree | Neither agree nor disagree | Somewhat agree | Agree |
| --- | --- | --- | --- | --- | --- |
| The main reason clinical trials of advanced therapies are conducted are to provide treatments for future patients | □ | □ | □ | □ | □ |
| Clinical trials of advanced therapies provide the best opportunity for current patients to access treatments for their disease | □ | □ | □ | □ | □ |
| Advanced therapies offer patients the best opportunity for a cure | □ | □ | □ | □ | □ |
| The risks of participating in a clinical trial of an advanced therapy that has not been tried in patients before outweighs the possible benefits | □ | □ | □ | □ | □ |
| The main reason clinical trials are conducted are to improve the treatment of patients on the trial. | □ | □ | □ | □ | □ |
| The benefits of participating in a clinical trial of an advanced therapy outweigh the possible risks | □ | □ | □ | □ | □ |
| I would not want my child to have an advanced therapy that had not been tried in humans before | □ | □ | □ | □ | □ |
| Advanced therapies will prevent a patient’s condition getting worse | □ | □ | □ | □ | □ |
| Patients may not benefit directly from participating in a clinical trial of an advanced therapy | □ | □ | □ | □ | □ |
| If there are no other therapies available, parents should be able to try unproven therapies for their child | □ | □ | □ | □ | □ |
| By participating in a clinical trial, patients will help the doctors and scientists learn information that may benefit that child | □ | □ | □ | □ | □ |
| By participating in a clinical trial, patients will help doctors and scientist learn information that may benefit future patients. | □ | □ | □ | □ | □ |
| An acceptable benefit of participating in a clinical trial is achieving stability | □ | □ | □ | □ | □ |
| An acceptable benefit of participating in a clinical trial is achieving symptom relief. | □ | □ | □ | □ | □ |

1. How adequate is the information available regarding advanced therapies (please tick)?

| □  Poor | □  Fair | □  Good | □  Very good | □  Excellent |
| --- | --- | --- | --- | --- |

1. Please rate your preferences for delivery of information regarding advanced therapies

| 1 being your most preferred and 6 being your least preferred. | | | |
| --- | --- | --- | --- |
|  | Internet resource with frequently asked questions (e.g. website) |  | In person seminar |
|  | Mobile application |  | Webinar |
|  | Printed resources (e.g. booklet) |  | Information from medical specialists |

1. Please rate your preferences for delivery of support regarding advanced therapies

| 1 being your most preferred and 6 being your least preferred | | | |
| --- | --- | --- | --- |
|  | Disease specific patient support groups |  | Support from doctors experienced with the advanced therapy |
|  | Internet resource with frequently asked questions |  | Support from government agency |
|  | No support |  | Support from psychologist or counsellor |

Please rank the sources of health information you trust the most.

| 1 being your most trusted and 6 being your least trusted | | | |
| --- | --- | --- | --- |
|  | Facebook disease groups run by parents |  | Government |
|  | Health professionals |  | News outlets |
|  | Disease specific websites |  | Friends and family |

Please select what types of diseases you believe should be the priority for new experimental interventions with uncertain risks and benefits? (select as few or as many as you like)

| Diseases that: |  |
| --- | --- |
| □ have no alternative therapies | □ have alternative therapies |
| □ have a low mortality | □ have a high mortality |
| □ have severe symptoms | □ have mild symptoms |
| □ get worse over time | □ stay the same |
| □ are at an early stage | □ are at an advanced stage |

Please rate how much you agree with the following statements about long-term follow-up and data sharing after an experimental intervention? Long-term follow-up means that the child attends the hospital for monitoring and medical tests after the experimental treatment has been administered.

|  | Disagree | Somewhat disagree | Neither agree nor disagree | Somewhat agree | Agree |
| --- | --- | --- | --- | --- | --- |
| It is not necessary for all children receiving an experimental advanced therapy to be followed up over time | □ | □ | □ | □ | □ |
| Following an experimental advanced therapy, all children should be followed up for a minimum of 5 years | □ | □ | □ | □ | □ |
| All children receiving an advanced neurotherapeutic intervention should be followed up into adulthood | □ | □ | □ | □ | □ |
| Data collected during experimental clinical trials of advanced therapies should be made available to the research community | □ | □ | □ | □ | □ |
