# Supplementary material for: ‘Advocacy groups are the connectors’: Experiences and contributions of rare disease patient organization leaders in advanced neurotherapeutics
Source: Health Expect. 2022 Oct 28;25(6):3175–91. doi: 10.1111/hex.13625 (PMC9700154; doi:10.1111/hex.13625)
Supplement: Supplementary file 4 — Supporting information. [file HEX-25--s004.docx]

**Appendix D:** DASS-21 Survey and Score Calculations (information retrieved from Lovibond and Lovibond39)

**DASS-21 Survey Questions**

Please read each statement and choose which indicates how much the statement applied to you over the past week.

1. I find it hard to wind down.

- Did not apply to me at all (0)
- Applied to some degree, or some of the time (1)
- Applied to me to a considerable degree or a good part of time (2)
- Applied to me very much or most of the time (3)

1. I was aware of dryness of my mouth.

- Did not apply to me at all (0)
- Applied to some degree, or some of the time (1)
- Applied to me to a considerable degree or a good part of time (2)
- Applied to me very much or most of the time (3)

1. I couldn't seem to experience any positive feeling at all.

- Did not apply to me at all (0)
- Applied to some degree, or some of the time (1)
- Applied to me to a considerable degree or a good part of time (2)
- Applied to me very much or most of the time (3)

1. I experienced breathing difficulty. (e.g. excessively rapid breathing, breathlessness in the absence of physical exertion)

- Did not apply to me at all (0)
- Applied to some degree, or some of the time (1)
- Applied to me to a considerable degree or a good part of time (2)
- Applied to me very much or most of the time (3)

1. I found it difficult to work up the initiative to do things.

- Did not apply to me at all (0)
- Applied to some degree, or some of the time (1)
- Applied to me to a considerable degree or a good part of time (2)
- Applied to me very much or most of the time (3)

1. I tended to over-react to situations.

- Did not apply to me at all (0)
- Applied to some degree, or some of the time (1)
- Applied to me to a considerable degree or a good part of time (2)
- Applied to me very much or most of the time (3)

1. I experienced trembling. (e.g. in the hands)

- Did not apply to me at all (0)
- Applied to some degree, or some of the time (1)
- Applied to me to a considerable degree or a good part of time (2)
- Applied to me very much or most of the time (3)

1. I felt that I was using a lot of nervous energy.

- Did not apply to me at all (0)
- Applied to some degree, or some of the time (1)
- Applied to me to a considerable degree or a good part of time (2)
- Applied to me very much or most of the time (3)

1. I was worried about situations in which I might panic and make a fool of myself.

- Did not apply to me at all (0)
- Applied to some degree, or some of the time (1)
- Applied to me to a considerable degree or a good part of time (2)
- Applied to me very much or most of the time (3)

1. I felt that I had nothing to look forward to.

- Did not apply to me at all (0)
- Applied to some degree, or some of the time (1)
- Applied to me to a considerable degree or a good part of time (2)
- Applied to me very much or most of the time (3)

1. I found myself getting agitated.

- Did not apply to me at all (0)
- Applied to some degree, or some of the time (1)
- Applied to me to a considerable degree or a good part of time (2)
- Applied to me very much or most of the time (3)

1. I found it difficult to relax.

- Did not apply to me at all (0)
- Applied to some degree, or some of the time (1)
- Applied to me to a considerable degree or a good part of time (2)
- Applied to me very much or most of the time (3)

1. I felt down-hearted and blue.

- Did not apply to me at all (0)
- Applied to some degree, or some of the time (1)
- Applied to me to a considerable degree or a good part of time (2)
- Applied to me very much or most of the time (3)

1. I was tolerant of anything that kept me from getting on with what I was doing.

- Did not apply to me at all (0)
- Applied to some degree, or some of the time (1)
- Applied to me to a considerable degree or a good part of time (2)
- Applied to me very much or most of the time (3)

1. I felt I was close to panic.

- Did not apply to me at all (0)
- Applied to some degree, or some of the time (1)
- Applied to me to a considerable degree or a good part of time (2)
- Applied to me very much or most of the time (3)

1. I was unable to become enthusiastic about anything.

- Did not apply to me at all (0)
- Applied to some degree, or some of the time (1)
- Applied to me to a considerable degree or a good part of time (2)
- Applied to me very much or most of the time (3)

1. I felt I wasn't worth much as a person.

- Did not apply to me at all (0)
- Applied to some degree, or some of the time (1)
- Applied to me to a considerable degree or a good part of time (2)
- Applied to me very much or most of the time (3)

1. I felt that I was rather touchy.

- Did not apply to me at all (0)
- Applied to some degree, or some of the time (1)
- Applied to me to a considerable degree or a good part of time (2)
- Applied to me very much or most of the time (3)

1. I was aware of the action of my heart in the absence of physical exertion. (e.g. sense of heart rate increase, heart missing a beat)

- Did not apply to me at all (0)
- Applied to some degree, or some of the time (1)
- Applied to me to a considerable degree or a good part of time (2)
- Applied to me very much or most of the time (3)

1. I felt scared without any good reason.

- Did not apply to me at all (0)
- Applied to some degree, or some of the time (1)
- Applied to me to a considerable degree or a good part of time (2)
- Applied to me very much or most of the time (3)

1. I felt that life was meaningless.

- Did not apply to me at all (0)
- Applied to some degree, or some of the time (1)
- Applied to me to a considerable degree or a good part of time (2)
- Applied to me very much or most of the time (3)

***Calculation for DASS-21 scores***

Each question response is allocated a value as follows: did not apply to me at all = 0; applied to some degree or some of the time = 1; applied to me to a considerable degree or a good part of the time = 2; applied to me very much or most of the time = 3. Each negative mood state is associated with specific questions, which are arithmetically summed and then multiplied by two^39^. Recommended cut-off ranges for normal, mild, moderate, severe, and extremely severe syndromes are outlined in the table below.

Table of recommended cut-off scores for conventional severity labels in depression, anxiety, and stress and associated DASS-21 survey questions (information taken from Lovibond and Lovibond^39^)

| **Assessed mood state** | **Questions included in sum** | **Normal range** | **Mild range** | **Moderate range** | **Severe range** | **Extremely severe range** |
| --- | --- | --- | --- | --- | --- | --- |
| Depression | 3, 5, 10, 13, 16, 17, 21 | 0-9 | 10-13 | 14-20 | 21-27 | 28+ |
| Anxiety | 2, 4, 7, 9, 15, 19, 20 | 0-7 | 8-9 | 10-14 | 15-19 | 20+ |
| Stress | 1, 6, 8, 11, 12, 14, 18 | 0-14 | 15-18 | 19-25 | 26-33 | 34+ |
