# Supplementary material for: ‘Advocacy groups are the connectors’: Experiences and contributions of rare disease patient organization leaders in advanced neurotherapeutics
Source: Health Expect. 2022 Oct 28;25(6):3175–91. doi: 10.1111/hex.13625 (PMC9700154; doi:10.1111/hex.13625)
Supplement: Supplementary file 5 — Supporting information. [file HEX-25--s005.docx]

**Appendix E**: Study recruitment strategy
